# Spermine reverses lipopolysaccharide-induced memory deficit in mice

**DOI:** 10.1186/s12974-014-0220-5

**Published:** 2015-01-09

**Authors:** Pâmella Karina Santana Frühauf, Rafael Porto Ineu, Lediane Tomazi, Thiago Duarte, Carlos Fernando Mello, Maribel Antonello Rubin

**Affiliations:** Graduation Program in Pharmacology, Center of Health Sciences, Federal University of Santa Maria, Santa Maria, RS 97105-900 Brazil; Department of Physiology and Pharmacology, Center of Health Sciences, Federal University of Santa Maria, Santa Maria, RS 97105-900 Brazil; Department of Biochemistry and Molecular Biology, Natural and Exact Sciences Center, Federal University of Santa Maria, Camobi, CEP: 97105900 Santa Maria, RS Brazil

**Keywords:** Ifenprodil, Memory, Neuroinflammation, NMDA receptor object recognition, Polyamines, Spermine

## Abstract

**Background:**

Lipopolysaccharide (LPS) induces neuroinflammation and memory deficit. Since polyamines improve memory in various cognitive tasks, we hypothesized that spermine administration reverses LPS-induced memory deficits in an object recognition task in mice. The involvement of the polyamine binding site at the N-methyl-D-aspartate (NMDA) receptor and cytokine production in the promnesic effect of spermine were investigated.

**Methods:**

Adult male mice were injected with LPS (250 μg/kg, intraperitoneally) and spermine (0.3 to 1 mg/kg, intraperitoneally) or ifenprodil (0.3 to 10 mg/kg, intraperitoneally), or both, and their memory function was evaluated using a novel object recognition task. In addition, cortical and hippocampal cytokines levels were measured by ELISA four hours after LPS injection.

**Results:**

Spermine increased but ifenprodil decreased the recognition index in the novel object recognition task. Spermine, at doses that did not alter memory (0.3 mg/kg, intraperitoneally), reversed the cognitive impairment induced by LPS. Ifenprodil (0.3 mg/kg, intraperitoneally) reversed the protective effect of spermine against LPS-induced memory deficits. However, spermine failed to reverse the LPS-induced increase of cortical and hippocampal cytokine levels.

**Conclusions:**

Spermine protects against LPS-induced memory deficits in mice by a mechanism that involves GluN2B receptors.

## Background

It has been shown that intraperitoneal injection of lipopolysaccharide (LPS), a cell-wall component of gram-negative bacteria, induces neuroinflammation, hippocampal cell loss, cognitive impairment, learning deficits and even β-amyloid plaque generation in the hippocampus [1,2], constituting a valid experimental model to study the physiological, behavioral, and emotional aspects of sickness behavior [3]. A number of studies have shown that upon exposure to LPS, microglia become activated and produce proinflammatory mediators such as cytokines, chemokines, prostanoids, and reactive oxygen species. Current evidence indicates that these products are key mediators of the neuroinflammatory process and contribute to LPS-induced neuronal damage and subsequent cognitive loss [1,4-8]. In line with this view, it has been shown that LPS administration impairs contextual fear conditioning [8-10] and spatial memory [2,11-14] and avoidance learning [15-19] in rats and mice. In addition, LPS decreases the preference for a novel object in a novel object recognition test in mice [20]. The comprehension of the mechanisms by which such a deficit occurs may unveil additional (or ratify already-known) regulatory mechanisms of memory consolidation and retrieval in this pathologic condition, thereby guiding the search for novel therapeutic strategies or compounds to mitigate these deficits.

The N-methyl-D-aspartate (NMDA) receptors seem to be particularly susceptible to the neuroinflammatory challenge, since inflammation decreases the expression of GluN1 [21], GluN2A and GluN2B [22] subunits, and NMDA-dependent long-term potentiation [23] in the hippocampus. This finding is in agreement with previous reports that neuroinflammation decreases total NMDA receptors (NR1 immunoreactivity) in the hippocampus and entorhinal cortex [24] and that LPS-treated animals present decreased MK801 binding [25]. Accordingly, the partial NMDA receptor agonist D-cycloserine prevents the deleterious effects of LPS [26] and closed head injury [27] on memory consolidation. Therefore, it sounds possible that other positive allosteric modulators of the NMDA receptor attenuate LPS-induced cognitive deficits.

Polyamines, such as putrescine, spermidine, and spermine, allosterically activate NMDA receptors by binding at the lower lobe of the N-terminal domain of GluN1 and GluN2B dimer interface [28]. Functionally, polyamines are involved in growth and differentiation, but they also regulate a broad array of cellular functions in both neurons and inflammatory cells [29-31]. Numerous reports have indicated that polyamines improve memory in several tasks and attenuate memory deficits induced by different amnesic agents [32-41]. In fact, agonists and antagonists of the polyamine binding site at the NMDA receptor respectively facilitate and impair memory in various tasks [33,34,36,39,40,42,43], and the sequential activation of protein kinase C (PKC) and PKA/CREB (protein kinase A/cAMP response element-binding protein) pathways in the hippocampus has been implicated in the promnesic effect of polyamines [44,45]. However, it has recently been described that interruption of the NMDA receptor modulation by polyamines reverses Aβ_25–35_-induced memory impairment in mice in a novel object recognition task [46], suggesting that the role of polyamines in memory may vary in physiological and pathological conditions.

The assumption that the effects of polyamines on memory depend on physiological or pathological conditions is in agreement with clinical and experimental data that support a beneficial role for NMDA antagonists in Alzheimer’s disease [46,47]. However, one might ask whether this applies to every pathological or neuroinflammatory condition. Therefore, we investigated the effect of spermine on LPS-induced impairment of memory in a novel object recognition test. In addition, we investigated whether spermine alters LPS-induced increase of cortical and hippocampal cytokine levels in mice, since both anti- and proinflammatory activity have also been reported for these aliphatic amines [29,48-51].

## Methods

### Animals

Adult male Swiss mice approximately 12 weeks old (30 to 35 g) provided by the Animal Center of Universidade Federal de Santa Maria, were used for the behavioral experiments. The animals had free access to water and food (Guabi, Santa Maria, Rio Grande do Sul, Brazil), and were maintained in a humidity- and temperature-controlled room (22 ± 2°C) with a 12-hour light-dark cycle. Behavioral experiments were conducted in a sound-attenuated and air-regulated room, where the animals were habituated for 1 hour prior to experiments. Behavioral tests were conducted during the light phase of the cycle (between 9:00 a.m. and 5:00 p.m.). All animal procedures were carried out in accordance with Brazilian law no. 11.794/2008, which is in agreement with the Policies on the Use of Animals and Humans in Neuroscience research, revised and approved by the Society for Neuroscience Research in January 1995 and with the Institutional and National regulations for animal research (process 068/2011). All experimental protocols were designed with the aim of keeping the number of animals used to a minimum, as well as their suffering.

### Drug administration

Lipopolysaccharide (*Escherichia coli*, serotype 055:B5), spermine (N, N′-bis (3-aminopropyl) 1,4-butanediamine) and ifenprodil (α-(4-hydroxyphenyl)-β-methyl- 4-benzyl-1-piperidineethanol tartrate salt) were obtained from Sigma (St. Louis, MO, USA). All drug solutions were prepared daily in saline (0.9% NaCl) and injections were performed intraperitoneally in a 10 ml/kg injection volume. Doses were selected based on previous studies [8], and pilot experiments.

### Behavioral testing

#### Novel object recognition task

A novel object recognition task was carried out as described previously [46]. The task was performed in a 30 × 30 × 30 cm wooden chamber, with walls painted black, a front wall made of Plexiglas and a floor covered with ethyl vinyl acetate sheet. A light bulb, hanging 60 cm above the behavioral apparatus, provided constant illumination of about 40 lux, and an air-conditioner provided constant background sound isolation. The objects used were plastic mounting bricks, each with different shapes and colors, but the same size. Throughout the experiments, objects were used in a counterbalanced manner. Animals had not previously displayed a preference for any of the objects. Chambers and objects were cleaned with 30% ethanol immediately before and at the end of each behavioral evaluation. The task consisted of habituation, training, and testing sessions, each lasting 8 minutes. In the first session, mice were individually habituated to the behavioral apparatus and then returned to their home cages. Twenty-four hours later, the animals were subjected to a training session in which the animals were exposed to two of the same objects (object A), and the exploration time was recorded with two stopwatches. Exploration was recorded when the animal touched or reached the object with the nose at a distance of less than 2 cm. Climbing or sitting on the object was not considered exploration. The test session was carried out 24 hours after training. Mice were placed back in the behavioral chamber and one of the familiar objects (object A) was replaced by a novel object (object B). The times spent exploring the familiar and the novel object were recorded. The discrimination index was then calculated, taking into account the difference of time spent exploring the new and familiar objects, using the formula:

$$ \left(\left[\left({T}_{\mathrm{novel}}-{T}_{\mathrm{familiar}}\right)/\left({T}_{\mathrm{novel}}+{T}_{\mathrm{familiar}}\right)\right]\times 100\left(\%\right)\right) $$

The discrimination index was used as a memory parameter.

#### Open field

Immediately after the object recognition test session, the animals were transferred to a 30 cm × 30 cm open field, with the floor divided into four squares. During the 5-min open field session, the number of crossing and rearing responses was recorded. The open field was used to identify motor disabilities, which might influence the object recognition performance.

#### Quantification of cytokines

Cytokine quantification was assessed by ELISA using commercial kits for mouse IFN-γ, TNF-α, IL-1β, IL-6, and IL-10 (eBIO-SCIENCE, San Diego, CA, USA), according to the manufacturer’s instructions. The presence and concentration of the cytokines were determined by the intensity of the color measured by spectrometry in a micro ELISA reader.

### Statistical analysis

Statistical analyses were performed using Student’s *t* test, one, two or three-way analysis of variance (ANOVA) followed by the Bonferroni post-hoc analysis. A value of *P* < 0.05 was considered significant. *F* and *P* values are shown only if *P* < 0.05.

### Experimental design

#### Experiment 1

This experiment was designated to investigate the effect of LPS on the object recognition task performance. Animals were habituated and trained, as described. Immediately after training, the animals were injected with saline or LPS (250 μg/kg). Twenty-four hours after training, the animals were subjected to the novel object recognition test session and open field as described.

#### Experiment 2

This experiment was designated to investigate the effect of spermine or ifenprodil on the object recognition task performance. Animals were habituated and trained as described. Immediately after training, the animals were injected with saline, spermine (0.1 to 10 mg/kg) or ifenprodil (0.3 to 10 mg/kg). Twenty-four hours after training, the animals were subjected to the novel object recognition test session and open field, as described.

#### Experiment 3

This experiment was designed to investigate the involvement of polyamine binding sites in the impairment of memory induced by LPS. Animals were habituated and trained as described. Immediately after training, the animals were injected with saline or LPS (250 μg/kg) and 5 minutes later with saline or the polyaminergic agonist, spermine (at doses that have no effect *per se* on memory, 0.3 mg/kg, as determined by the dose-effect curve in different flanks. Twenty-four hours after training, the animals were subjected to the novel object recognition test session and open field, as described.

#### Experiment 4

This experiment was designed to investigate the involvement of polyamine binding sites on the NMDA receptor in the reversal of the LPS-induced impairment of memory by spermine on the object recognition task performance. Animals were habituated and trained as described. Immediately after training, the animals were injected with saline or LPS (250 μg/kg), 5 min later they were injected with saline or the polyaminergic agonist, spermine (0.3 mg/kg, the dose that reverses the LPS-induced impairment of memory), and after another 5 minutes they were injected with saline or ifenprodil (at doses that have no effect *per se* on memory, 0.3 mg/kg, as determined by the dose-effect curve) in different flanks. Twenty-four hours after training, the animals were submitted to the novel object recognition test and open field, as described.

#### Experiment 5

This experiment was designed to investigate whether spermine prevents LPS-induced increases in levels of proinflammatory cytokines, IL-1β, IL-6, TNF-α, interferon-γ (ITF-γ), and anti-inflammatory cytokine, IL-10. Immediately after the training session of the object recognition task, animals received saline, LPS (250 μg/kg), spermine (0.3 mg/kg) or a combination of LPS (250 μg/kg) and spermine (0.3 mg/kg). Four hours after drug administration, the animals were anesthetized with ketamine (100 mg/kg) and xylazine (10 mg/kg) and transcardially perfused with cold saline. The cerebral cortex and the hippocampi were dissected and homogenized in appropriated buffer (PBS containing 1 mM ethylenediamine tetraacetic acid (EDTA), 0.1 mM phenylmethylsulfonyl fluoride and 0.5% BSA, pH 7.4). Samples were centrifuged at 25,000 *g* for 10 min and the supernatant was used to measure IL-1β, IL-6, TNF-α, ITF-γ, and IL-10 levels, which were corrected for total protein content. Protein concentration was determined for each brain region by the Bradford method [52] enabling cytokine levels to be expressed as pg/mg protein. The cytokine quantification was assessed as described.

## Results

### LPS decreased the recognition index in novel object recognition task

**Figure 1:**
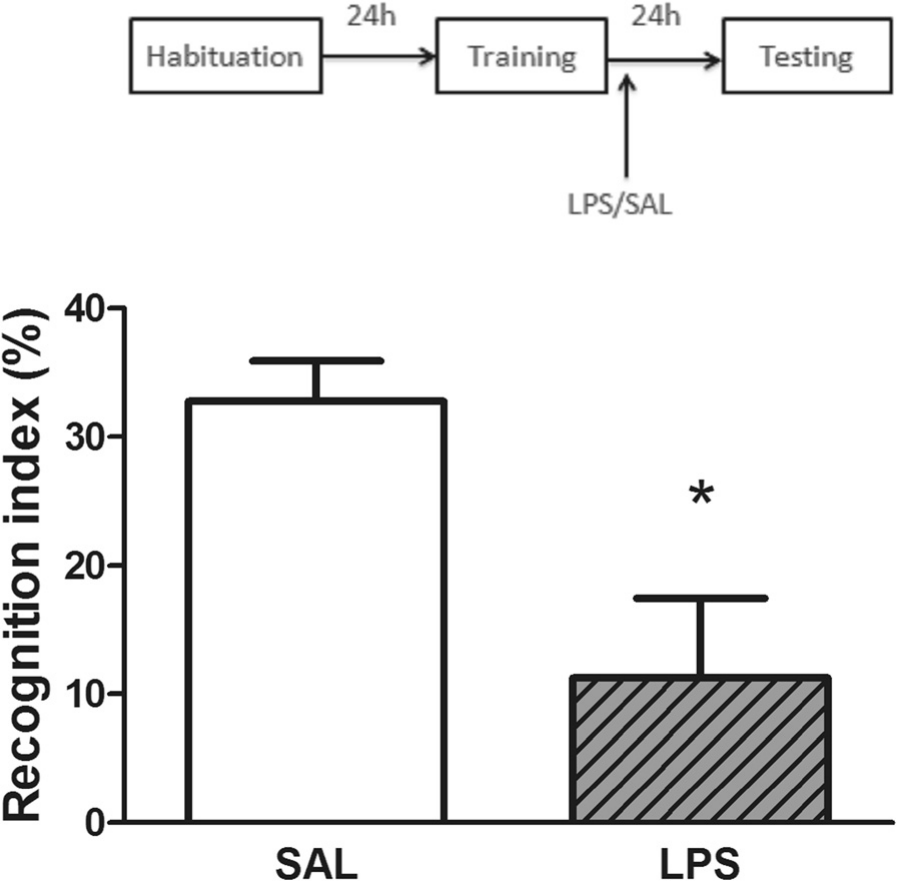
**Effects of LPS on memory.** Post-training administration of LPS (250 μg/kg, intraperitoneally) decreased the recognition index in the object recognition task. Data are expressed as mean ± standard error of the mean for 8 animals in each group. *P < 0.01 compared with saline group (Student’s *t* test). LPS, lipopolysaccharide; SAL, saline.

Figure 1 shows the effect of the post-training intraperitoneal administration of LPS or saline on recognition index in the object recognition task. Statistical analysis (Student’s *t* test) revealed that LPS (250 μg/kg) decreased the recognition index (*t*_14_ = 3.086; *P* < 0.01; *η*^2^ = 0.4), compared with the control group, indicating a memory impairment in LPS-treated animals.

### Spermine increased and ifenprodil decreased the recognition index on novel object recognition task

**Figure 2:**
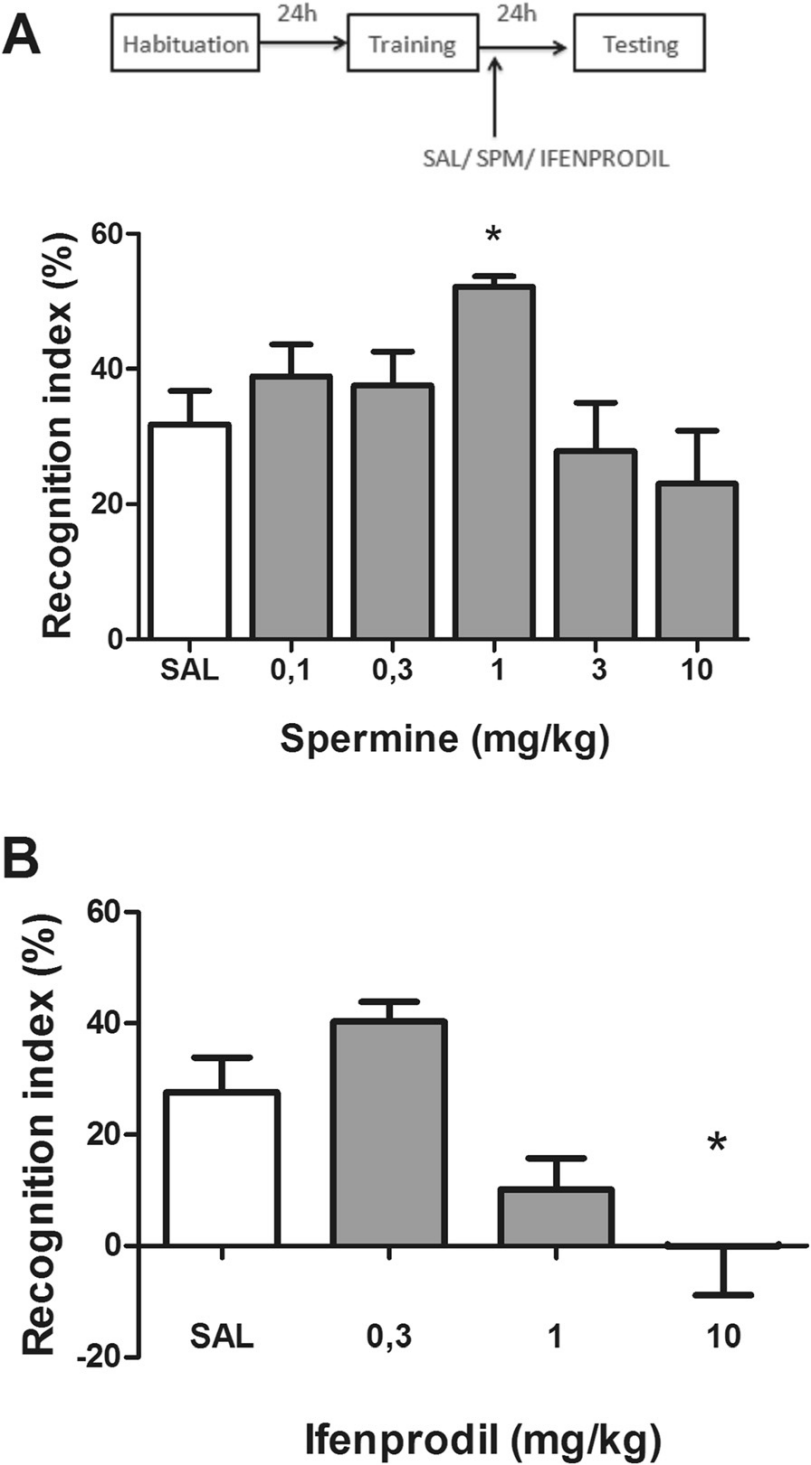
**Effect of administration of spermine or ifenprodil on memory.** Immediately after training, the animals were injected with saline, spermine (0.1 to 10 mg/kg, intraperitoneally) or ifenprodil (0.3 to 10 mg/kg, intraperitoneally). Twenty-four hours after training, the animals were subjected to the novel object recognition test session. **(A)** Spermine increased and **(B)** ifenprodil decreased the recognition index on novel object recognition task. Date are expressed as mean ± standard error of the mean for 6 to 9 animals in each group. *P < 0.01 compared with saline group (one-way ANOVA followed by the Bonferroni’s post-hoc test). SAL, saline; SPM, spermine.

Figure 2A shows the effect of post-training administration of spermine (0.1 to 10 mg/kg) on the recognition index in the object recognition task. Statistical analysis (one-way ANOVA) revealed a significant effect of spermine (*F*_5,44_ = 3.76; *P* < 0.05; *η*^2^ = 0.29). Post-hoc analysis revealed that spermine (1 mg/kg) increased the recognition index, indicating that spermine improved memory.

Figure 2B shows the effect of post-training administration of ifenprodil (0.3 to 10 mg/kg) on the recognition index in the object recognition task. Statistical analysis (one-way ANOVA) revealed a significant effect of ifenprodil (*F*_3,17_ = 7.28; *P* < 0.005; *η*^2^ = 0.56). Post-hoc analysis revealed that ifenprodil (10 mg/kg) decreased the recognition index, indicating that ifenprodil impaired memory.

### Spermine attenuates LPS-induced discrimination impairments on novel object recognition task

**Figure 3:**
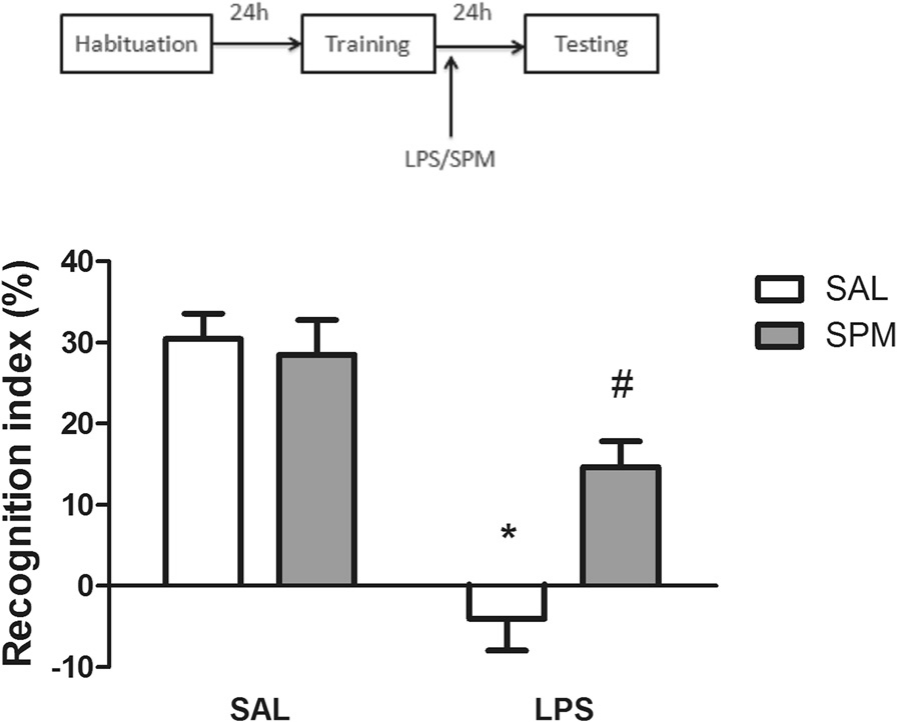
**Effect of spermine in LPS-induced memory impairment.** Post-training administration of spermine (0.3 mg/kg, intraperitoneally) attenuates LPS-induced (250 μg/kg, intraperitoneally) discrimination impairments on novel object recognition task. Data are expressed as mean ± standard error of the mean for 15 animals in each group. *P < 0.005 compared with saline group, #*P* < 0.005 compared with LPS and saline group (two-way ANOVA followed by the Bonferroni post-hoc test). LPS, lipopolysaccharide; SAL, saline; SPM, spermine.

Figure 3 shows the effect of post-training administration of spermine on LPS-induced impairment of memory in the novel object recognition task. Statistical analysis (two-way ANOVA) revealed a significant treatment (saline or LPS) versus polyamine agonist (saline or spermine) interaction (*F*_1,56_ = 8.10; *P* < 0.01; η^2^ = 0.12), revealing that spermine reversed the impairment of memory induced by LPS.

### Ifenprodil reverses the effect of spermine in LPS-treated animals

**Figure 4:**
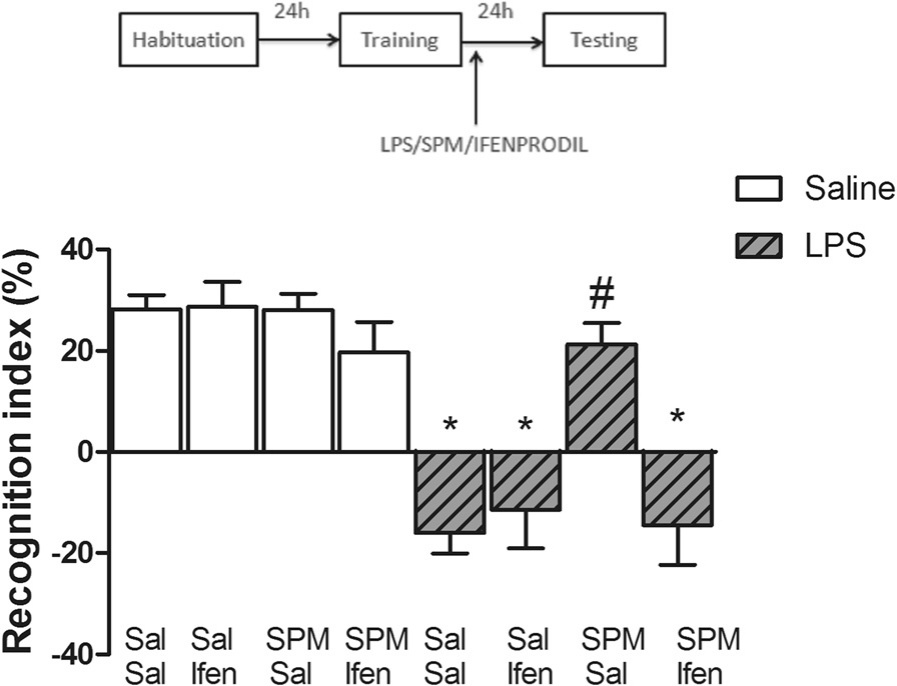
**Effect of agonist or antagonist of the NMDA receptor on LPS-treated animal’s memory.** Immediately after training, the animals were injected with saline or LPS (250 μg/kg, intraperitoneally), 5 min later they were injected with saline or spermine (0.3 mg/kg, intraperitoneally), and 5 minutes after that they were injected with saline or ifenprodil (0.3 mg/kg, intraperitoneally) in different flanks. Ifenprodil reversed the effect of spermine in LPS-treated animals. Data are the mean ± standard error of the mean of 13 to 15 animals per group. *P < 0.05 compared with control group Sal/Sal/Sal, #*P* < 0.05 compared with LPS/Sal/Sal group (three-way ANOVA followed by the Bonferroni post-hoc test). Ifen, ifenprodil; LPS, lipopolysaccharide; Sal, saline; SPM, spermine.

Figure 4 shows the effect of ifenprodil (0.3 mg/kg) on the spermine-induced reversal of the impairment of memory induced by LPS in the object recognition task. Statistical analysis (three-way ANOVA) revealed a significant treatment (saline or LPS) versus polyamine agonist (saline or spermine) versus NMDA receptor antagonist (saline or ifenprodil) interaction (*F*_1,110_ = 4.45; *P* < 0.05; *η*^2^ = 0.038). Post-hoc analysis (Bonferroni) revealed that ifenprodil reversed the effect of spermine in attenuating the impairment of memory induced by LPS on the novel object recognition task.

### Treatments did not alter crossing responses.

Since the recognition index might be affected by locomotor alterations unrelated to the mnemonic component of the task, we monitored the number of crossing responses in the open field task. Treatments did not alter locomotor activity measured by crossing responses (data not shown). Therefore, the reported effects in this study are unlikely to be associated with changes in locomotion and coordination.

### Spermine did not reverse LPS-induced increase of proinflammatory cytokines levels

**Figure 5:**
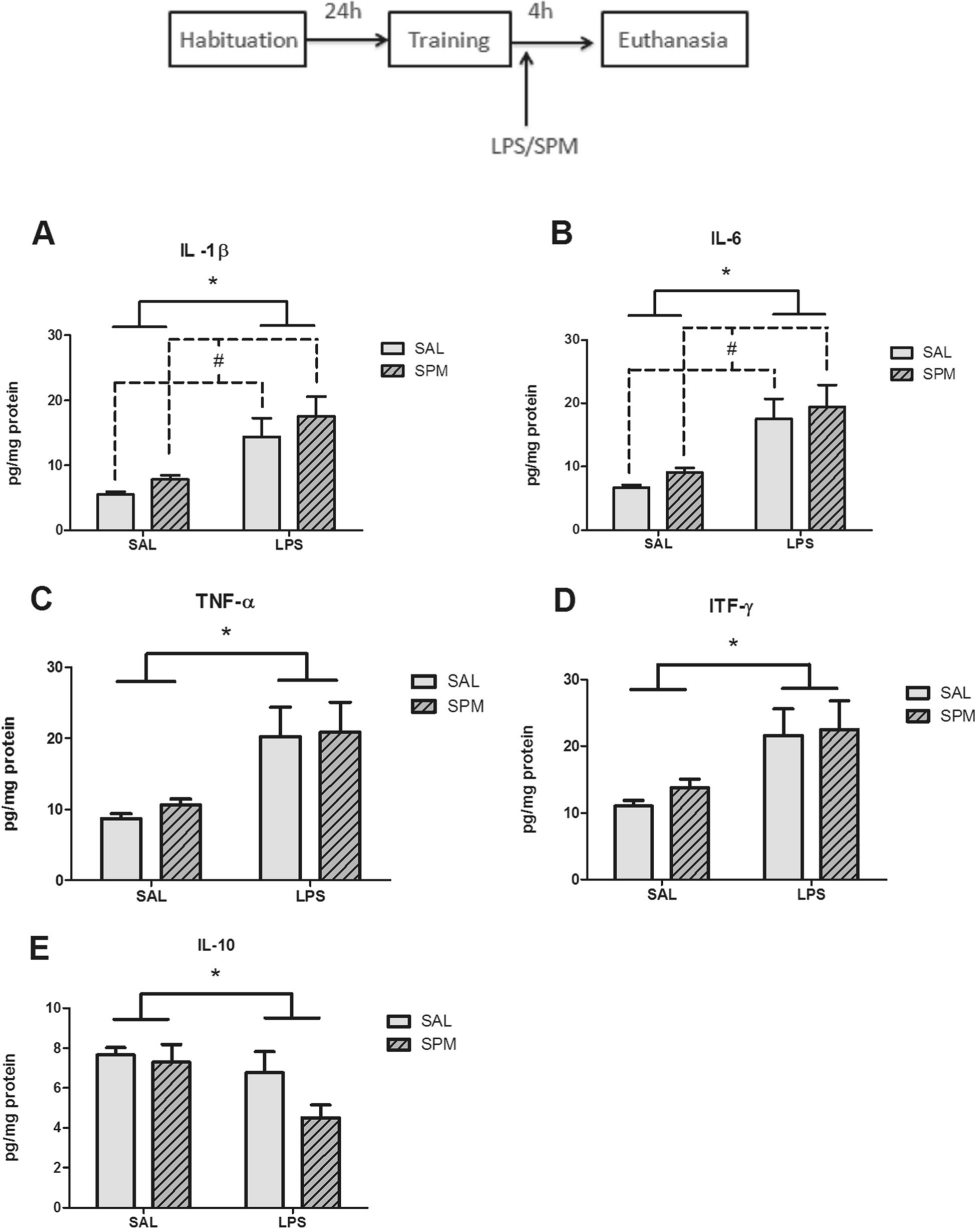
**Effects of post-training administration of spermine and LPS in the levels of proinflammatory and anti-inflammatory cytokines in hippocampus.** Immediately after the training session of the object recognition task, animals received saline, LPS (250 μg/kg, intraperitoneally), spermine (0.3 mg/kg, intraperitoneally) or a combination of LPS (250 μg/kg, intraperitoneally) and spermine (0.3 mg/kg, intraperitoneally), and were euthanized 4 hours after injections to measure the levels of **(A)** IL-1β, **(B)** IL-6, **(C)** TNF-α, **(D)** ITF-γ, and **(E)** IL-10 in the hippocampus. *P < 0.05 between pooled post-training saline (SAL-SAL and SAL-SPM groups) and pooled post-training LPS (LPS-SAL and LPS-SPM groups) by Bonferroni’s *t* test. #*P* < 0.05 between pooled post-training saline (SAL-SAL and LPS-SAL groups) and pooled post-training spermine (SAL-SPM and LPS-SPM groups). Data are the mean ± standard error of the mean of 4 or 5 animals per group. LPS, lipopolysaccharide; SAL, saline; SPM, spermine.

**Figure 6:**
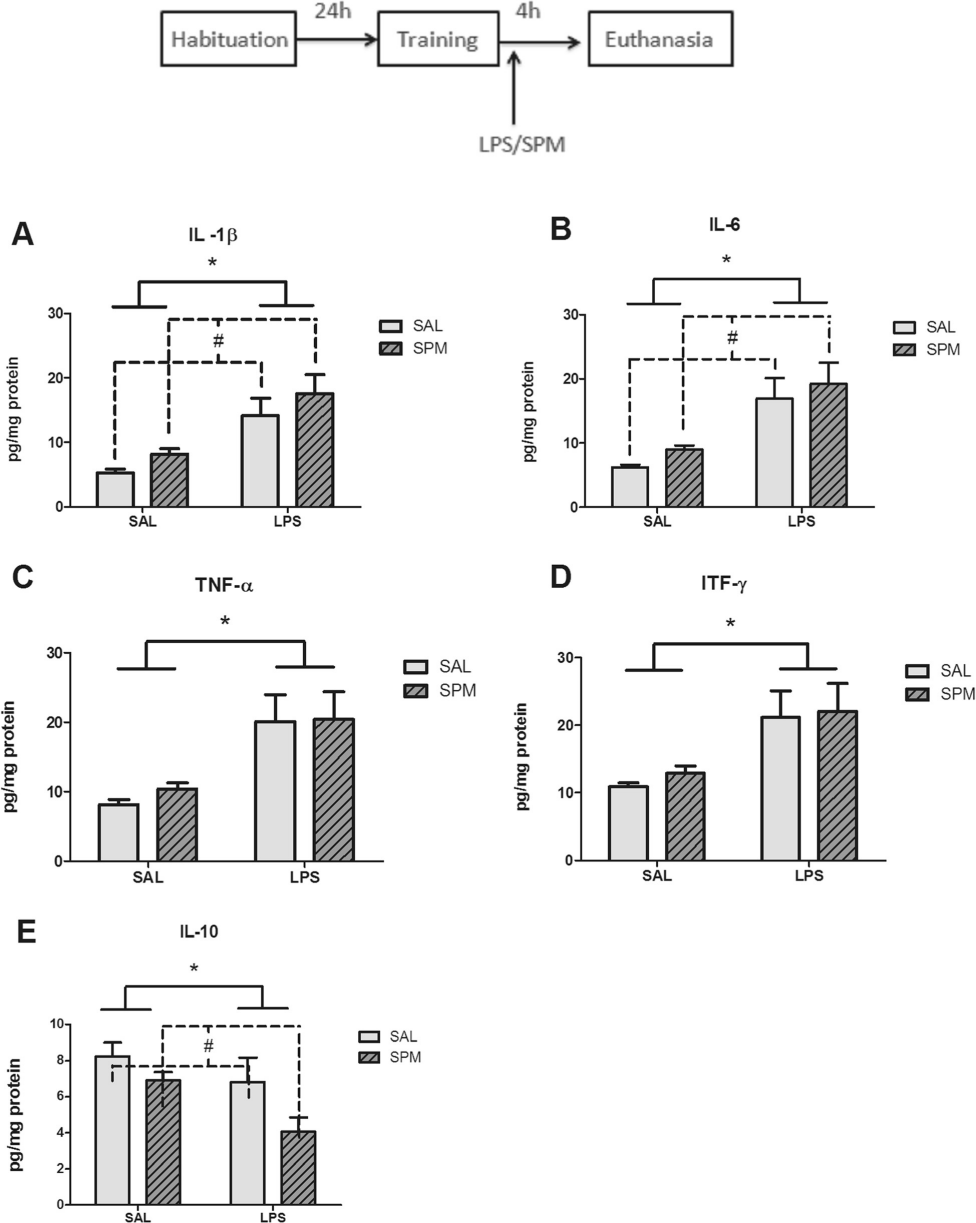
**Effects of post-training administration of spermine and LPS in the levels of proinflammatory and anti-inflammatory cytokines in cerebral cortex.** Immediately after the training session of the object recognition task, animals received saline, LPS (250 μg/kg, intraperitoneally), spermine (0.3 mg/kg, intraperitoneally) or a combination of LPS (250 μg/kg, intraperitoneally) and spermine (0.3 mg/kg, intraperitoneally), and were euthanized 4 hours after injections to measure the levels of **(A)** IL-1β, **(B)** IL-6, **(C)** TNF-α, **(D)** ITF-γ, and **(E)** IL-10 in the cerebral cortex. * *P* < 0.05 between pooled post-training saline (SAL-SAL and SAL-SPM groups) and pooled post-training LPS (LPS-SAL and LPS-SPM groups) by Bonferroni’s *t* test. #*P* < 0.05 between pooled post-training saline (SAL-SAL and LPS-SAL groups) and pooled post-training spermine (SAL-SPM and LPS-SPM groups). Data are the mean ± standard error of the mean of 4 or 5 animals per group. LPS, lipopolysaccharide; SAL, saline; SPM, spermine.

Figures 5 and 6 show the effect of post-training administration of spermine and LPS in the levels of proinflammatory and anti-inflammatory cytokines in the hippocampus and cerebral cortex respectively. Statistical analysis (two-way ANOVA) revealed a significant effect of treatment (saline or LPS) on IL-1β (*F*_1,15_ = 40.01; *P* < 0.001; *η*^2^ = 0.72), IL-6 (*F*_1,15_ = 52.43; *P* < 0.001; *η*^2^ = 0.77), TNF-α (*F*_1,15_ = 27.64; *P* < 0.001; *η*^2^ = 0.64) and IL-10 (*F*_1,15_ = 9.26; *P* < 0.01; *η*^2^ = 0.38) levels in the hippocampus (Figure 5), and on IL-1β (*F*_1,15_ = 37.74; *P* < 0.001; *η*^2^ = 0.71), IL-6 (*F*_1,15_ = 47.43; *P* < 0.001; *η*^2^ = 0.75) and TNF-α (*F*_1,15_ = 30.43; *P* < 0.001; *η*^2^ = 0.67), IL-10 (*F*_1,5_ = 8.78; *P* < 0.05; *η*^2^ = 0.36) levels in the cerebral cortex (Figure 6). Both LPS and spermine alter the levels of IL-1β (*F*_1,15_ = 4.78; *P* < 0.05; *η*^2^ = 0.24), IL-6 (*F*_1,15_ = 4.92; *P* < 0.05; *η*^2^ = 0.24), and IL-10 (*F*_1,15_ = 4.76; *P* < 0.05; *η*^2^ = 0.24) levels in the hippocampus (Figure 5), and on IL-1β (*F*_1,15_ = 6.14; *P* < 0.05; *η*^2^ = 0.29), IL-6 (*F*_1,15_ = 4.92; *P* < 0.05; *η*^2^ = 0.24) and IL-10 (*F*_1,5_ = 5.95; *P* < 0.05; *η*^2^ = 0.28) levels in the cerebral cortex (Figure 6).

## Discussion

This study showed that a polyaminergic agonist, spermine, reverses post-training bacterial endotoxin-induced memory impairment in a novel object recognition task. In addition, it showed that spermine, at doses higher than those capable of reversing the deleterious effect of LPS, increases the recognition index in the novel object recognition task. It also revealed that ifenprodil decreases the recognition index in the novel object recognition task, and a non-effective dose of ifenprodil reverses the memory-improving effect of spermine in LPS-treated animals, providing pharmacological evidence that the promnesic effect of spermine involves the polyamine binding site at the NMDA receptor. However, spermine failed to reverse the LPS-induced increase of cytokine levels in the cerebral cortex or hippocampus.

The currently described ability of spermine to reverse LPS-induced memory deficits is in agreement with a number of studies that have separately shown that while LPS disrupts [1,8,13,15,53], polyamines improve memory [32-39,54].

Several mechanisms have been implicated in LPS-induced alterations of neural functions. It has been suggested that memory impairment is triggered by direct stimulation of toll-like receptor 4 (TLR_4_) by LPS. This TLR_4_ activation recruits myeloid differentiation adaptor protein (MyD88) and ultimately activates the nuclear factor κB signaling pathway, increasing the production of proinflammatory cytokines by macrophages [55], microglia [56-58] and astrocytes [56,59]. The LPS-induced activation of glial cells results in the release of neurotoxic substances, including nitric oxide, glutamate, cytotoxic cytokines, and superoxide radicals [8,56,60,61], the suppression of neurotrophic factor secretion, and impaired neuroplasticity and, consequently, learning and memory [8,9,11,17,62,63]. In addition, TLR_4_ activates Src family kinases, which phosphorylate GluN2B subunit or NMDA receptor and enhance GluN2B-dependent Ca^2+^ influx [64,65], promoting excitotoxicity. Indeed, LPS induces progressive and cumulative neuronal loss over time [66-68].

Conversely, there is a large body of evidence indicating that the promnesic effects of polyamines involve the activation of NMDA receptors, which have been critically implicated in learning and memory processes [36,40,44,45,69-71]. In the current study, ifenprodil, a noncompetitive GluN2B-containing NMDA receptor antagonist, not only impaired the consolidation of the memory of novel object recognition task, but also reversed (at a dose that did not alter memory *per se* in our study) the improving effect of spermine on the memory of LPS-treated animals, supporting a role for NMDA receptors in this effect of spermine [72-74]. Interestingly, these results are in agreement with the study by Kranjac and colleagues [26], who have shown that partial NMDA receptor agonist D-cycloserine rescues memory consolidation following systemic bacterial endotoxin exposure. Furthermore, Velloso and colleagues [41] have found that post-training intrastriatal administration of spermine reverses the recognition memory deficits in the novel object recognition task induced by quinolinic acid, a model of Huntington’s disease. It is worth noting that spermine improved memory *per se* in the novel object recognition task. To our knowledge, this is the first study showing that spermine improves memory. The finding that ifenprodil prevents the promnesic effect of spermine tempts us to propose that it may involve the same molecular targets proposed for spermidine [33,34,36,38]. However, further studies are necessary to clarify this point.

We have found that while LPS increased IL1-β, IL-6, TNF-α, and IFN-γ levels, it decreased IL-10 levels in the cerebral cortex and hippocampus. This is in agreement with previous studies that have shown that systemic administration of this endotoxin causes neuroinflammation [56-58]. Moreover, spermine increased IL1-β and IL-6 levels in the cerebral cortex and hippocampus *per se*, indicating that it facilitates inflammation, but to a much lesser degree than LPS. Interestingly, spermine also decreased the anti-inflammatory cytokine IL-10 in both cerebral structures, further supporting a proinflammatory role for this polyamine in our experimental conditions. These results are in line with other studies [51,75] which show that the spermine facilitates inflammation in *vivo*. Accordingly, it has been shown that polyamines promote macrophage influx into the murine central nervous system following pathogenic insult, in an *in vivo* model of secondary central nervous system inflammation [51]. Soulet and Rivest [75] have shown that systemic LPS increases ornithine decarboxylase expression throughout the central nervous system and that this precedes an increase in the expression of proinflammatory molecules TLR_2_ and TNF-α, in mice. Moreover, treatment with a difluoromethylornithine, an irreversible inhibitor of ornithine decarboxylase, decreases LPS-induced TNF-α and TLR_2_ expression, proving evidence that the LPS-induced increase of proinflammatory molecules is polyamine-dependent. The intracerebral injection of spermine prior to LPS also increases the number of cells expressing both TNF-α and TLR_2_ in the central nervous system [75]. Interestingly, LPS exposure stimulates IL-1β release from astrocytes (*in vitro*) through a mechanism that requires NMDA receptor stimulation [76].

We hypothesized that polyamines could decrease LPS-induced cognitive impairment by interfering in cytokine levels because: (1) LPS, at doses that cause cognitive impairment, sequentially increases brain IL-6, IL-1β, and TNF-α mRNA levels in the hippocampus [77,78] and frontal cortex [79]; (2) LPS-induced upregulation of IL-1β and TNF-α mRNA in hippocampal tissue of IL-6^(+/+)^ mice is absent in IL-6^(−/−)^ mice, which are also refractory to the LPS-induced impairment in working memory [77]. In addition, overexpression of TNF-α in neurons or glial cells impairs passive avoidance memory [80]; (3) Pharmacological treatments that decrease the levels of TNF-α, TNF-α receptor 1, and NF-κB p65 phosphorylation also decrease LPS-induced memory deficits [81,82], though conventional TNF^(−/−)^ knockout mice present cognitive dysfunction [83,84]. A recent review summarized the effect of TNF-α, IL-6, and IL-1 on learning and memory [85], where the authors suggest that TNF-α and its receptors might mediate the disrupting effect of LPS on learning and memory. Therefore, considering the existing evidence supporting a role for cytokines, particularly TNF-α, on LPS-induced cognitive impairment, we thought that the memory-improving effects of spermine could involve a decrease of cytokine levels in the hippocampus.

It has recently been described that LPS reduces the number of excitatory synapses in the hippocampus and cerebral cortex, leading to synaptic deficits [86] that may underlie LPS-induced cognitive deficits. In fact, LPS induces neurotoxic substance release and suppression of neurotrophic factors secretion, which are known to increase neuroplasticity and, consequently, learning and memory. Since polyamines increase excitatory activity [28], it is possible that spermine actions involve a compensatory increase of excitatory transmission in LPS-treated animals. However, specific studies have to be performed, to clarify this point.

## Conclusions

Spermine improves memory *per se* and attenuates LPS-induced memory impairment. Our results also suggest that spermine protects against LPS-induced memory impairment by mechanisms that involve the polyamine binding site at the NMDA receptor, since it is reversed by ifenprodil. Spermine, however, does not prevent LPS-induced increase of proinflammatory molecules, suggesting that the effect of spermine on memory does not involve anti-inflammatory mechanisms.

## Abbreviations

ANOVA: analysis of variance
BSA: bovine serum albumin
CREB: cAMP response element-binding protein
EDTA: ethylenediamine tetraacetic acid
ELISA: enzyme-linked immunosorbent assay
IL-1β: interleukin-1β
IL-6: interleukin-6
IL-10: interleukin-10
ITF-γ: interferon-γ
LPS: lipopolysaccharide
MyD88: myeloid differentiation adaptor protein
NMDA: N-methyl-D-aspartate
PBS: phosphate buffered saline
PKA: protein kinase A
PKC: protein kinase C
TNF-α: tumor necrosis factor-α
TLR: toll-like receptor
